# Facile Synthesis of Functionalized Porous Carbon by Direct Pyrolysis of *Anacardium occidentale* Nut-Skin Waste and Its Utilization towards Supercapacitors

**DOI:** 10.3390/nano13101654

**Published:** 2023-05-16

**Authors:** Raji Atchudan, Suguna Perumal, Ashok K. Sundramoorthy, Devaraj Manoj, Raju Suresh Kumar, Abdulrahman I. Almansour, Yong Rok Lee

**Affiliations:** 1 School of Chemical Engineering, Yeungnam University, Gyeongsan 38541, Republic of Korea; 2 Department of Chemistry, Sejong University, Seoul 143747, Republic of Korea; suguna.perumal@gmail.com; 3 Department of Prosthodontics, Saveetha Dental College and Hospitals, Saveetha Institute of Medical and Technical Sciences, Poonamallee High Road, Velappanchavadi, Chennai 600077, Tamil Nadu, India; ashok.sundramoorthy@gmail.com; 4 Department of Chemistry, Karpagam Academy of Higher Education, Coimbatore 641021, Tamil Nadu, India; manojdvrj@gmail.com; 5 Centre for Material Chemistry, Karpagam Academy of Higher Education, Coimbatore 641021, Tamil Nadu, India; 6 Department of Chemistry, College of Science, King Saud University, Riyadh 11451, Saudi Arabia; sraju@ksu.edu.sa (R.S.K.); almansor@ksu.edu.sa (A.I.A.)

**Keywords:** *Anacardium occidentale*, biomass waste, porous carbon, pyrolysis, supercapacitor, environmentally friendly

## Abstract

Preparing electrode materials plays an essential role in the fabrication of high-performance supercapacitors. In general, heteroatom doping in carbon-based electrode materials enhances the electrochemical properties. Herein, nitrogen, oxygen, and sulfur co-doped porous carbon (PC) materials were prepared by direct pyrolysis of *Anacardium occidentale* (AO) nut-skin waste for high-performance supercapacitor applications. The as-prepared AO-PC material possessed interconnected micropore/mesopore structures and exhibited a high specific surface area of 615 m^2^ g^−1^. The Raman spectrum revealed a moderate degree of graphitization of AO-PC materials. These superior properties of the as-prepared AO-PC material help to deliver high specific capacitance. After fabricating the working electrode, the electrochemical performances including cyclic voltammetry, galvanostatic charge–discharge, and electrochemical impedance spectroscopy measurements were conducted in 1 M H_2_SO_4_ aqueous solution using a three-electrode configuration for supercapacitor applications. The AO-PC material delivered a high specific capacitance of 193 F g^−1^ at a current density of 0.5 A g^−1^. The AO-PC material demonstrated <97% capacitance retention even after 10,000 cycles of charge–discharge at the current density of 5 A g^−1^. All the above outcomes confirmed that the as-prepared AO-PC from AO nut-skin waste via simple pyrolysis is an ideal electrode material for fabricating high-performance supercapacitors. Moreover, this work provides a cost-effective and environmentally friendly strategy for adding value to biomass waste by a simple pyrolysis route.

## 1. Introduction

Carbon materials, especially porous carbons, are among the most promising electrode materials for energy storage applications due to their high and tunable specific surface area, chemical stability, comparatively low cost, rapid charge capability, outstanding electrical conductivity, substantial mechanical properties, and outstanding recyclability [1,2,3]. Hence, researchers have made considerable efforts to develop carbon-based electrodes for high-performance energy storage devices. Currently, energy storage devices mainly consist of zinc-air batteries, lithium-ion/sodium-ion batteries, liquid-flow batteries, supercapacitors, etc. [4,5]. Among energy storage devices, supercapacitors are considered attractive due to their advantages of low cost, superior power output efficiency, fast charge–discharge rates, long-cycle stability, higher energy density, light-weight, low maintenance, wide range of temperatures, and so on [6,7,8,9]. Supercapacitors can be classified into two main categories based on the energy storage mechanism of electrode materials: electric double-layer (EDL) capacitors and pseudocapacitors [4,10]. Energy is stored through electrostatic accumulation of charges at the electrode–electrolyte interfaces in EDL capacitors and by fast Faradaic reactions between the electrode and electrolyte [11]. Supercapacitors typically consist of three major parts: (i) electrolyte, (ii) electrode, and (iii) separator. The electrode materials play an essential role in super capacitive performance. Carbon-based materials including carbon nanotubes, carbon aerogels, and activated carbon are mainly applied as EDL capacitor electrodes owing to their extraordinary specific surface area and large pore structure [12,13,14]. However, the performance of regular carbon materials present some limitations to the EDL mechanism and they are unable to meet the increasingly demanding requirements [15]. Introducing heteroatoms such as nitrogen, phosphorus, sulfur, boron, and so on into the carbon skeleton is expected to be one of the most widespread tactics for enhancing the electrochemical performance of carbon materials towards their application in supercapacitors by providing additional pseudocapacitive characteristics to the EDL characteristics of the carbon materials [16,17].

Furthermore, heteroatom doping (nitrogen, phosphorus, sulfur, boron, etc.) into the carbon skeleton could enhance the surface wettability and electrical conductivity (decreasing the charge transfer resistance of carbon electrodes) of carbon materials, facilitating their electrochemical performance [18]. Nevertheless, the synthetic route is complicated, and some carbonaceous sources are expensive, delaying porous carbon use in supercapacitor applications. Therefore, biomass-derived porous carbon materials have been widely utilized as effective precursors for the preparation of supercapacitors due to the self-doping of heteroatoms, green resources, low cost, and so on [2]. These biomass-derived carbon materials possess a large specific surface area and have thus been employed as electrodes for supercapacitor applications [19,20,21,22,23,24]. Moreover, several heteroatom-doped porous carbons display better electrochemical performance than single-heteroatom-doped porous carbon due to the synergism between the heteroatoms and the porous carbon framework [25]. However, researchers are still focused on the development or tuning of carbon materials for supercapacitor applications. 

Hepsiba et al. successfully utilized *Anacardium occidentale* (AO) shell-derived porous carbon as the electrode material for supercapacitor applications [26]. In this study, AO nut-skin waste was used as a precursor to prepare heteroatom-doped porous carbon by direct pyrolysis. The resulting AO nut-skin waste-derived porous is denoted as AO-PC, exhibited multiple-heteroatom (nitrogen and sulfur) in the carbon skeleton and displayed a high specific surface area with suitable pores for electrochemical performances. After structural confirmation, as-prepared AO-PC material was employed as an electrode material for supercapacitor performance in 1 M H_2_SO_4_ aqueous electrolyte by a three-electrode configuration. The electrochemical performance was examined using cyclic voltammetry (CV), galvanostatic charge–discharge (GCD), and cycle life measurements. The as-prepared AO-PC material delivered excellent supercapacitive properties, and these results were compared to existing literature reports.

## 2. Experimental Section

### Preparation of AO-PC Materials

**Scheme 1:**
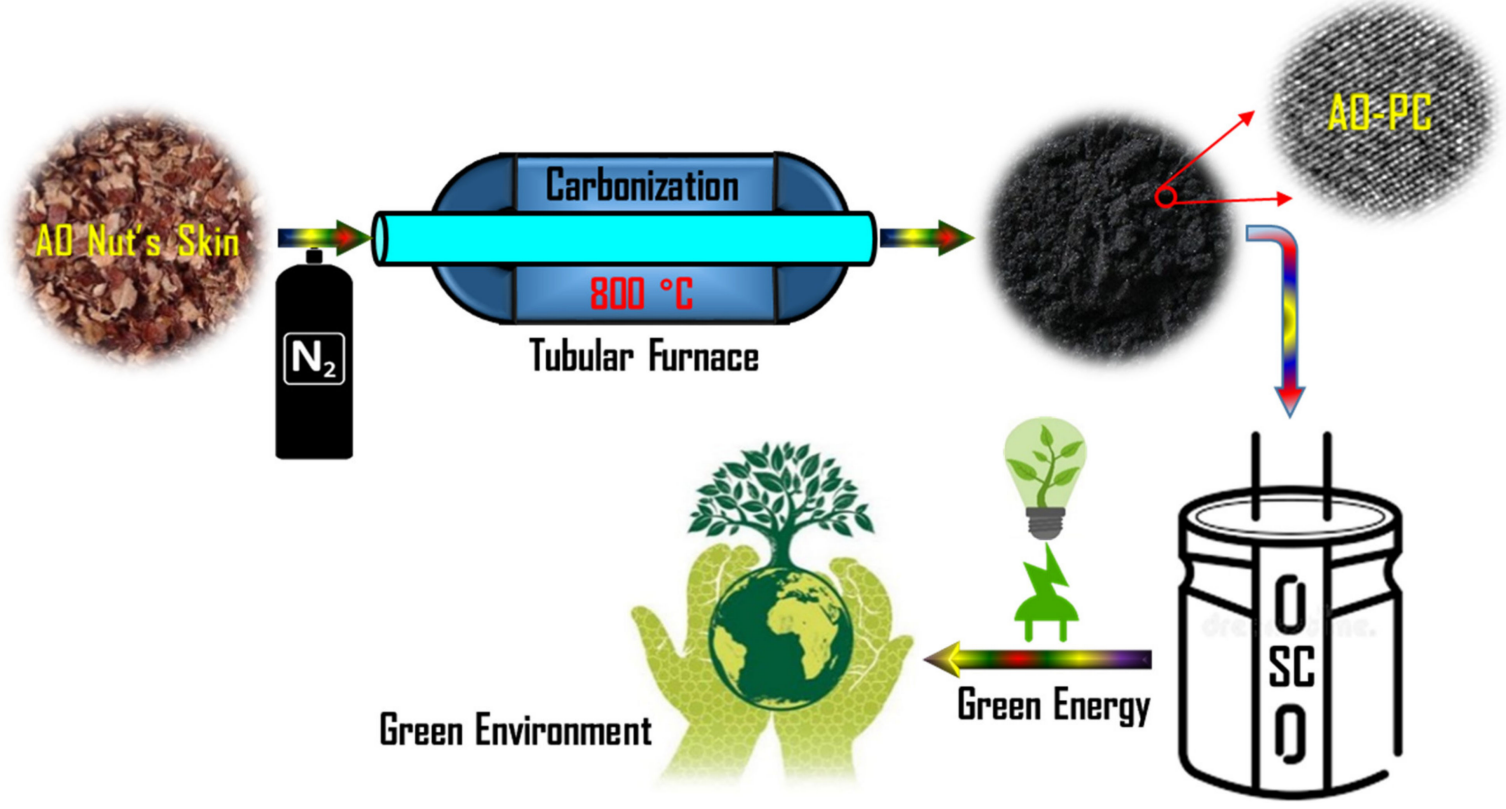
Schematic illustration of synthesis and application of dried *Anacardium occidentale* nut-skin waste-derived porous carbon.

AO-PC materials were prepared by direct pyrolysis as shown in Scheme 1. Specifically, *Anacardium occidentale* nut skins were ground well to make a fine powder using a commercial mixer and blender. The powdered raw material was taken into a quartz boat and kept in the middle of the quartz tube in the tubular furnace, and then the raw material was carbonized for 3 h at 800 °C in the presence of nitrogen gas. After the desired time, the furnace was cooled down to room temperature, and then the black powder was collected. Finally, the obtained black powder was ground well to make a homogenous slurry, which was denoted as AO-PC and used for the characterization and application. 

## 3. Results and Discussion

### 3.1. Structural Characterizations of as-Prepared AO-PC Material

**Figure 1:**
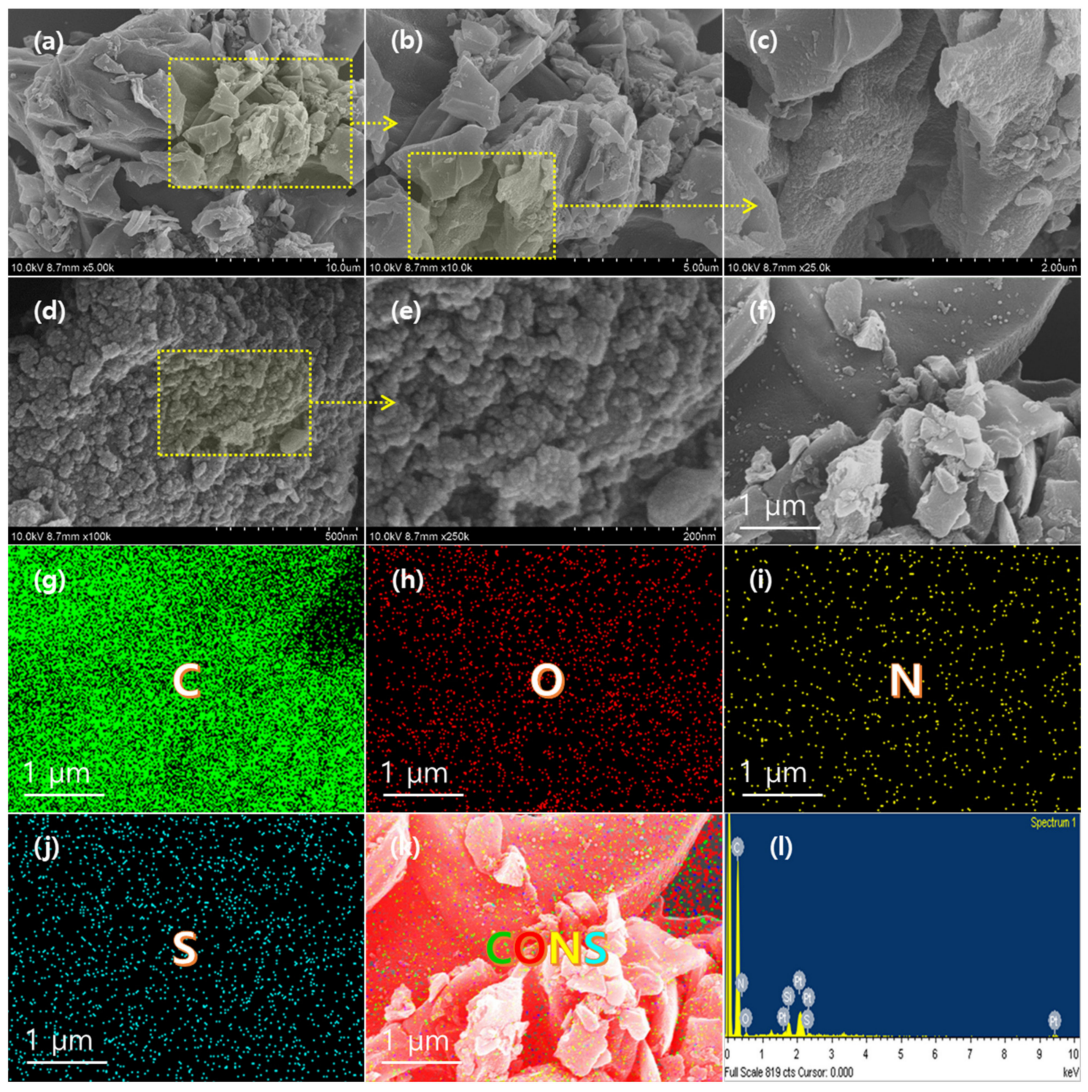
(**a**–**e**) FE-SEM images of the as-prepared AO-PC materials at different magnifications. (**f**) FE-SEM image with corresponding elemental mapping (**g**–**k**), and (**l**) EDX spectrum of as-prepared AO-PC material.

Field emission–scanning electron microscopy (FE-SEM) was employed to investigate the morphology and structure of the as-prepared AO-PC materials, and the corresponding FE-SEM of AO-PC materials is shown in Figure 1. Figure 1a–e show that the as-prepared AO-PC materials have a smooth surface morphology. Additionally, the as-prepared AO-PC materials show a small aggregation of smaller particles/sheets in the range of tens of nanometers. These are composed of porous sponge-like structures with irregular shapes and complex surface morphologies. It can be seen in the higher magnification the microstructure of the as-prepared AO-PC material is porous with the presence of many randomly interconnected macropores and mesopores that present a different range of diameters and shapes. Furthermore, the elemental composition of the as-prepared AO-PC material was revealed through an energy-dispersive X-ray (EDX) mapping and EDX spectral analysis. Figure 1f–k display the FE-SEM image and the corresponding EDX mapping of as-prepared AO-PC materials is displayed as Figure 1l. The EDX mapping shows that the as-prepared AO-PC materials contains carbon, oxygen, nitrogen, and sulfur elements. The elemental mapping also displays the excellent distribution of oxygen, nitrogen, and sulfur elements through the whole carbon matrix. The overlapping of mapping morphology (Figure 1k) indicates the presents the elements carbon, oxygen, nitrogen, and sulfur, and that they were distributed uniformly. The EDX spectrum (Figure 1l) also indicates that the as-prepared AO-PC material was mainly composed of carbon, oxygen, nitrogen, and sulfur elements with atomic ratios of 66, 28, 4, and 2%, respectively. The predominance of the carbon, oxygen, nitrogen, and sulfur elements strongly supports the absence of other impurities in the as-prepared AO-PC material. Apart from these carbon, oxygen, nitrogen, and sulfur elements, the EDX spectrum shows some elemental peaks including silicon and platinum, which might have originated from the silicon wafer substrate used for sample preparation and during the platinum sputter coating before EDX spectroscopic analysis, respectively.

**Figure 2:**
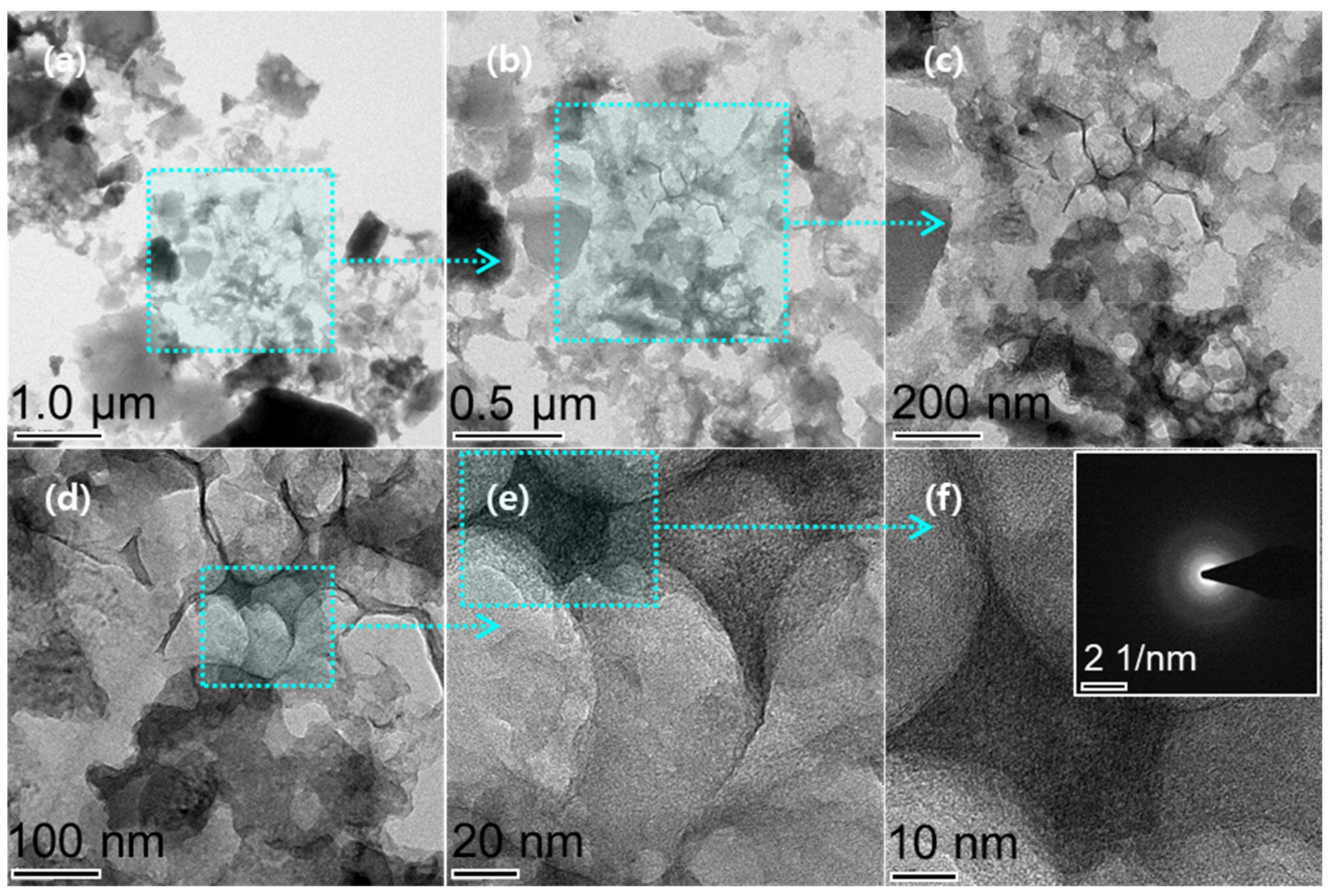
(**a**–**d**) TEM images and (**e**,**f**) HR-TEM images with different magnifications of the as-prepared AO-PC material (inset (**f**): SAED patterns of the as-prepared AO-PC material).

Furthermore, for a clear understanding of the morphology of the AO-PC materials, transmission electron microscopy (TEM), and high-resolution TEM (HR-TEM) were employed. The TEM images with different magnifications (Figure 2a–d) demonstrated that the as-prepared AO-PC material possessed a porous sponge-like structure. This porous structure might be due to the loosely arranged carbon lattice. The HR-TEM image of the as-prepared AO-PC material (Figure 2e,f) shows inconsistent lattice fringes, suggesting that the AO-PC material has a moderate degree of graphitization (partially amorphous in the carbon matrix). The inset of Figure 2f shows the selected area electron diffraction (SAED) pattern for the AO-PC material. The SAED pattern demonstrates that the as-prepared AO-PC material exhibits little diffuse diffraction pattern, suggesting a moderate degree of graphitization. This moderate degree of graphitization is due to the partially disordered surface/edges as well as the well-interconnected porosities of their carbon matrix. Thus, this porosity is believed to facilitate the penetration and transportation of electrons in energy storage applications [27].

**Figure 3:**
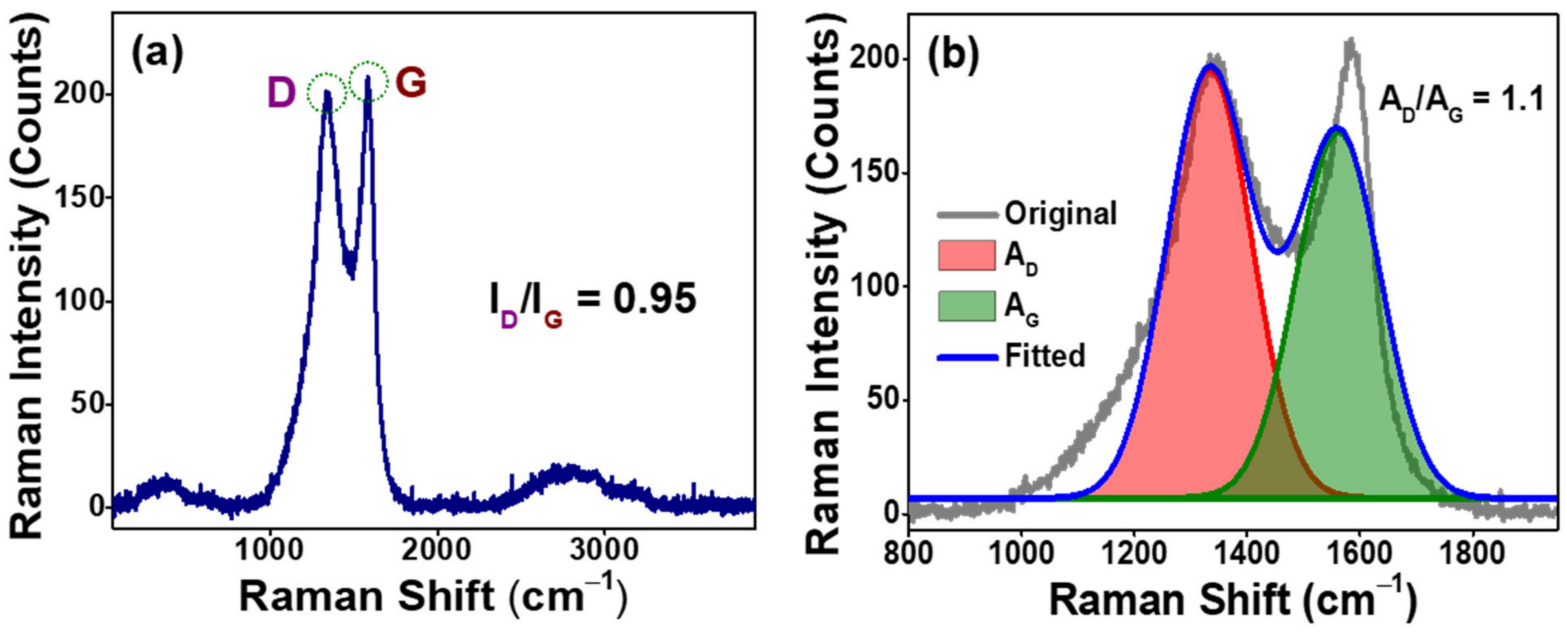
(**a**) Raman spectrum and (**b**) the corresponding deconvoluted Raman spectrum of the as-prepared AO-PC material.

To determine the degree of crystallinity/graphitization and phase purity of the as-prepared AO-PC material, XRD analysis was employed. As shown in Figure S1, the XRD pattern exhibited two prominent diffraction peaks centered at (2θ=) 24.5 and 43.5°, correlated with the scattering of the (0 0 2) and (1 0 0) crystal planes of typical carbon materials, respectively [24,28]. The slightly broad diffraction peaks revealed that the as-prepared AO-PC material had undergone a moderate degree of graphitization due to the amorphous structure (turbostratic-disturbed carbon structure) on the surface [29,30]. Apart from these prominent diffraction peaks, the absence of significant peaks indicates the purity of the AO-PC material. Raman spectroscopy was employed to further identify the structural features/nature of the materials including the degree of graphitization and purity of the as-prepared AO-PC material. Raman spectral studies of the as-prepared AO-PC material were carried out in the wavenumber range between 50 and 4000 cm^−1^, as shown in Figure 3a. The Raman spectrum displays two predominant bands centered at 1345 and 1585 cm^−1^, conforming to the D and G bands, respectively [31,32,33]. The D band originates from the disordered carbon (sp^3^-hybridized carbon) structures and the presence of structural defects such as edge imperfections, messy alignment, lattice defects, and low-symmetry graphitic structure in the prepared carbon materials [34,35]. However, the G band is associated with graphitic carbon structures and signifies the stretching vibration of sp^2^-hybridized carbon atoms (C=C stretching vibrations) in the carbon framework. The broader full width at half maximum (FWHM) of the D band compared to the G band suggests a higher disorder of sp^3^ carbon in the carbon framework [36]. This disordered nature might be due to the presence of abundant functional groups in AO-PC. Generally, the D-to-G-band ratio (I_D_/I_G_) determines the graphitization of the carbon materials [37]. Here, the I_D_/I_G_ ratio was calculated as 0.95, reflecting a good graphitization with a partially amorphous portion in the as-prepared AO-PC material, resulting in a moderate degree of graphitization. Note that there is no clear separation between the D and G bands. Because of the unclear separation, it is harder to judge the degree of graphitization of the AO-PC materials. Hence, the degree of graphitization of the as-prepared AO-PC material was determined through the area ratio of the D to the G bands (A_D_/A_G_) by deconvolution of the Raman spectrum, as shown in Figure 3b. Undoubtedly, the distinct and slightly greater area of the D band (the A_D_/A_G_ ratio is 1.1) indicates a moderate degree of carbon ordering in the AO-PC material. Apart from these two prominent peaks (1345 and 1585 cm^−1^), the broad fuzzy band centered around 2700 and 2930 cm^−1^ (between 2400 and 3300 cm^−1^) corresponds to the second-order D band (2D band) and the S_3_ band, respectively. These 2D and S_3_ bands are associated with the stacking of carbon layers (overtone of carbon) and reveal the presence of few-layered small carbon materials with a moderate graphitic nature [34,38]. Overall, these results indicate that the as-prepared AO-PC material exhibited a reasonable degree of graphitization with partial structural defects. 

The porosity and surface area of the prepared material are considered significant factors for supercapacitor applications. The as-prepared AO-PC material was investigated using nitrogen adsorption–desorption isotherms based on the Brunauer–Emmett–Teller (BET) surface area analysis regarding its porous structure and surface area characteristics. The BET adsorption–desorption curves of the as-prepared AO-PC material show type IV isotherms. The steep growth at low relative pressure (P/P_0_ < 0.2) indicates the presence of micropores. The broad hysteresis loop in the P/P_0_ range between 0.2 and 1.0 divulges the existence of a massive amount of mesopores [39]. The remarkable graph up-tick at high P/P_0_ (>0.2) implies type IV isotherms with an H3 hysteresis loop. Moreover, the type IV isotherm is recognized for the multilayer adsorption accompanying capillary condensation in tapered slit-like pores. The BET surface area of the as-prepared AO-PC material was found to be 615 m^2^ g^−1^ from the BET sorption isotherms. The high BET surface area of the as-prepared AO-PC material is due to their mesoporous nature. The pore distribution of the as-prepared AO-PC material was determined using the Barrett–Joyner–Halenda (BJH) method, and was in the range of 1.85–3.85 nm, with an average pore diameter of 3.5 nm (Figure S2). The pores exhibited by the as-prepared AO-PC material were mostly mesopores, suggesting the possibility of electrolyte ions passing through. Moreover, the high surface area and large pores are beneficial for energy storage and rate capability [40,41].

**Figure 4:**
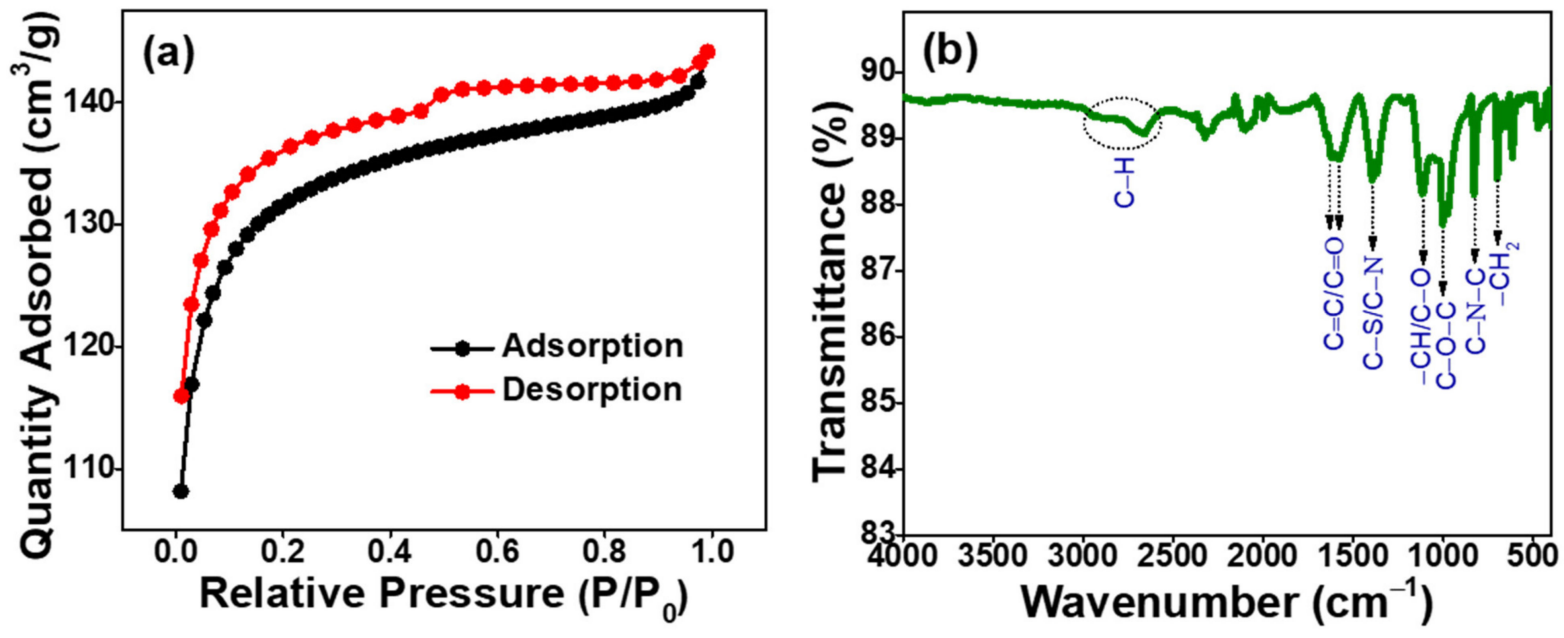
(**a**) Nitrogen adsorption–desorption curves and (**b**) ATR-FTIR spectrum of as-prepared AO-PC material.

As-prepared AO-PC material’s chemical composition and functional groups were investigated using attenuated total reflectance (ATR)–Fourier-transform infrared (FTIR) spectroscopy. In Figure 4b, the absorption bands at 2920 and 2850 cm^−1^ are responsible for the C–H (CH_2_/CH_3_) asymmetric and symmetric stretching vibrations, respectively. The well-resolved bands are visible in the IR spectrum at 1630, 1580, and 1393 cm^−1,^ which can be assigned to C=O (from the carbonyl and carboxylate functional groups), C=C (from the C=C bonds in the aromatic ring) and C–S/C–N (C–N^+^ ring-stretching vibrations) bonds, respectively [42,43]. The absorption peak at 1116 cm^−1^ can be attributed to the intra-surface –C–H/C–O vibrations, and the absorption peak at 1008 cm^−1^ can be ascribed to the C–O–C/–SO^3−^ bonds [44]. The bands at 830 and 700 cm^−1^ can be attributed to the C−N−C (wagging vibrations) and out-plane –CH_2_/–CH_3_ stretching vibrations, respectively [42,45]. A sharp peak at 617 cm^−1^ demonstrates the presence of the S−O bond from the sulfate ions in the as-prepared AO-PC material [46]. This examination reveals that the as-prepared AO-PC material possessed overwhelming functionalities on its surface/edges.

**Figure 5:**
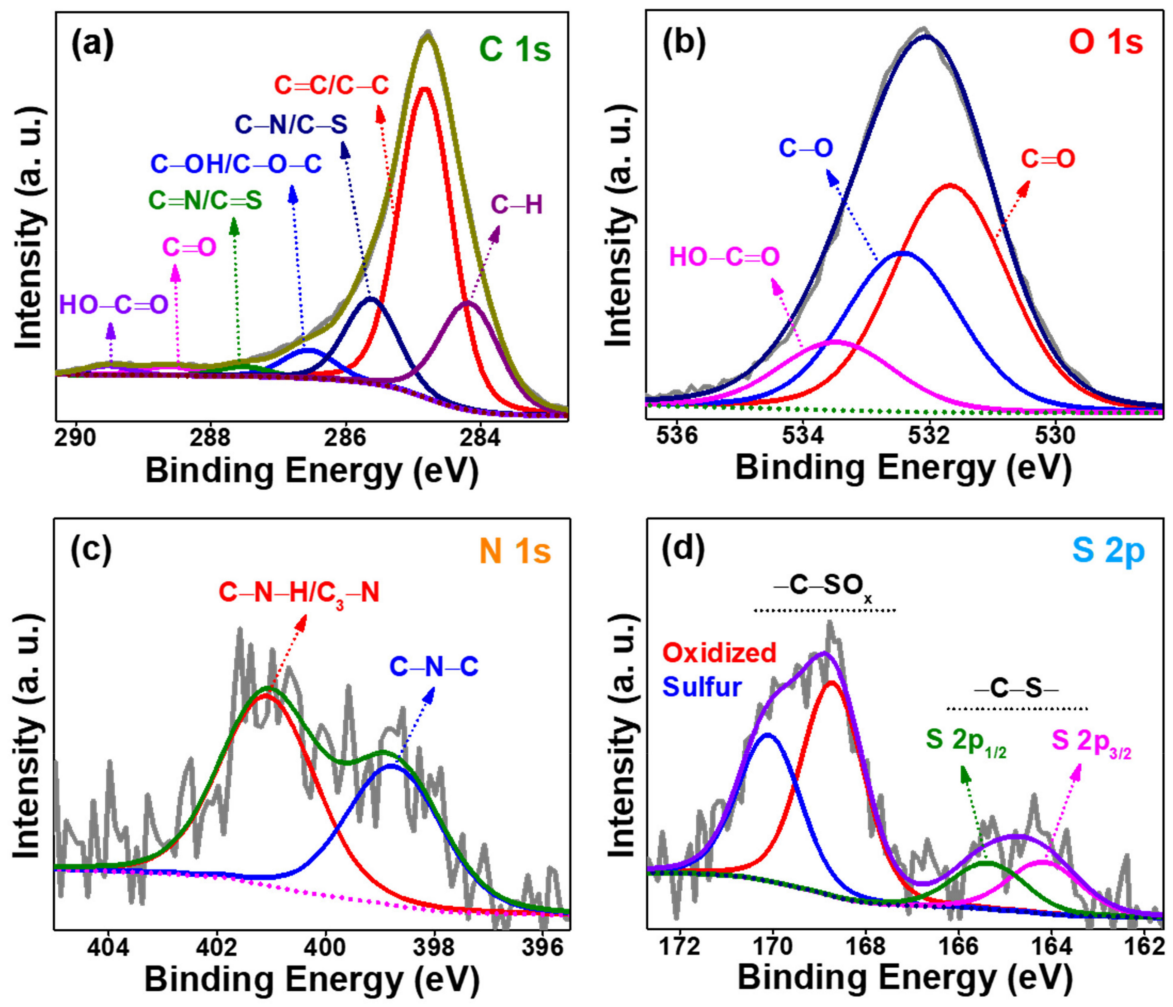
High-resolution XPS spectra: (**a**) C 1s, (**b**) O 1s, (**c**) N 1s, and (**d**) S 2p level of as-prepared AO-PC material.

XPS analysis was also conducted to determine the functional groups, elemental state, and chemical composition of the AO-PC. The XPS survey spectrum of the as-prepared AO-PC material is shown in Figure S3. The AO-PC material exhibits the characteristic peaks of S 2p, C 1s, N 1s, and O 1s located at ~165, 285, 401, and 532 eV, respectively, suggesting the presence of sulfur, carbon, nitrogen, and oxygen. The sulfur and nitrogen doping level in the as-prepared AO-PC material is around 1.5 and 3.5 atomic percentages, respectively. The high-resolution spectra of the as-prepared AO-PC material at S 2p, C 1s, N 1s, and O 1s levels are shown in Figure 5. The C 1s spectrum (Figure 5a) can be deconvoluted into seven individual component peaks centered at 284.2, 284.8, 285.6, 286.6, 287.6, 288.6, and 289.6 eV are assigned to C–H, C–C/C=C (sp^3^-C/sp^2^-C hybridization), C–N/C–S, C–OH/C–O–C, C=N/C=S, C=O, and O=C–OH, respectively, indicating the effective doping of sulfur, nitrogen and oxygen heteroatoms into the carbon framework [47,48]. The deconvoluted O 1s spectrum (Figure 5b) exhibited three prominent peaks around 531.7, 532.4, and 533.5 eV, which are responsible for the C=O, C–OH/C–O–C, and O=C–OH bonds, respectively [28,49]. The deconvoluted N 1s spectrum (Figure 5c) discerned two individual peaks centered at 398.9 and 401.1 eV that can be assigned to pyridinic nitrogen (C–N–C) and pyrrolic nitrogen (C–N–H)/quaternary N (graphitic nitrogen, C_3_–N), which are characteristically observed in nitrogen-doped carbon-based materials [44,50]. Generally, pyridinic and pyrrolic nitrogen contributes to the enhancement of the strong affinity of carbon atoms due to synergistic effects, resulting in good wettability and conductivity [51]. Nitrogen doping in the as-prepared AO-PC material could promote ion transport from the electrolyte to the electrode material, and may effectively enhance the capacitive properties [52]. The S 2p spectrum (Figure 5d) on deconvolution presented four peaks located at 164.2, 165.4, 168.7, and 170.1 eV. The 164.2 and 165.4 eV peaks correspond to –S–C/S–S bonds, and because of their spin-orbit couplings, they split into two components S 2p_3/2_ and S 2p_1/2_, respectively [52,53]. The 168.7 and 170.1 eV peaks are related to the oxidized sulfur species (–C–SO_x_) with different oxidation levels (x = 2 and 4) [54]. This suggests that sulfur doping in AO-PC material can enhance energy storage by increasing the pseudocapacitance of the electrode. This indicates that the sulfur, nitrogen, and oxygen atoms were effectively doped into the carbon framework of the as-prepared AO-PC material, which is in good agreement with the ATR-FTIR results.

### 3.2. Electrochemical Studies of as-Prepared AO-PC Material

**Figure 6:**
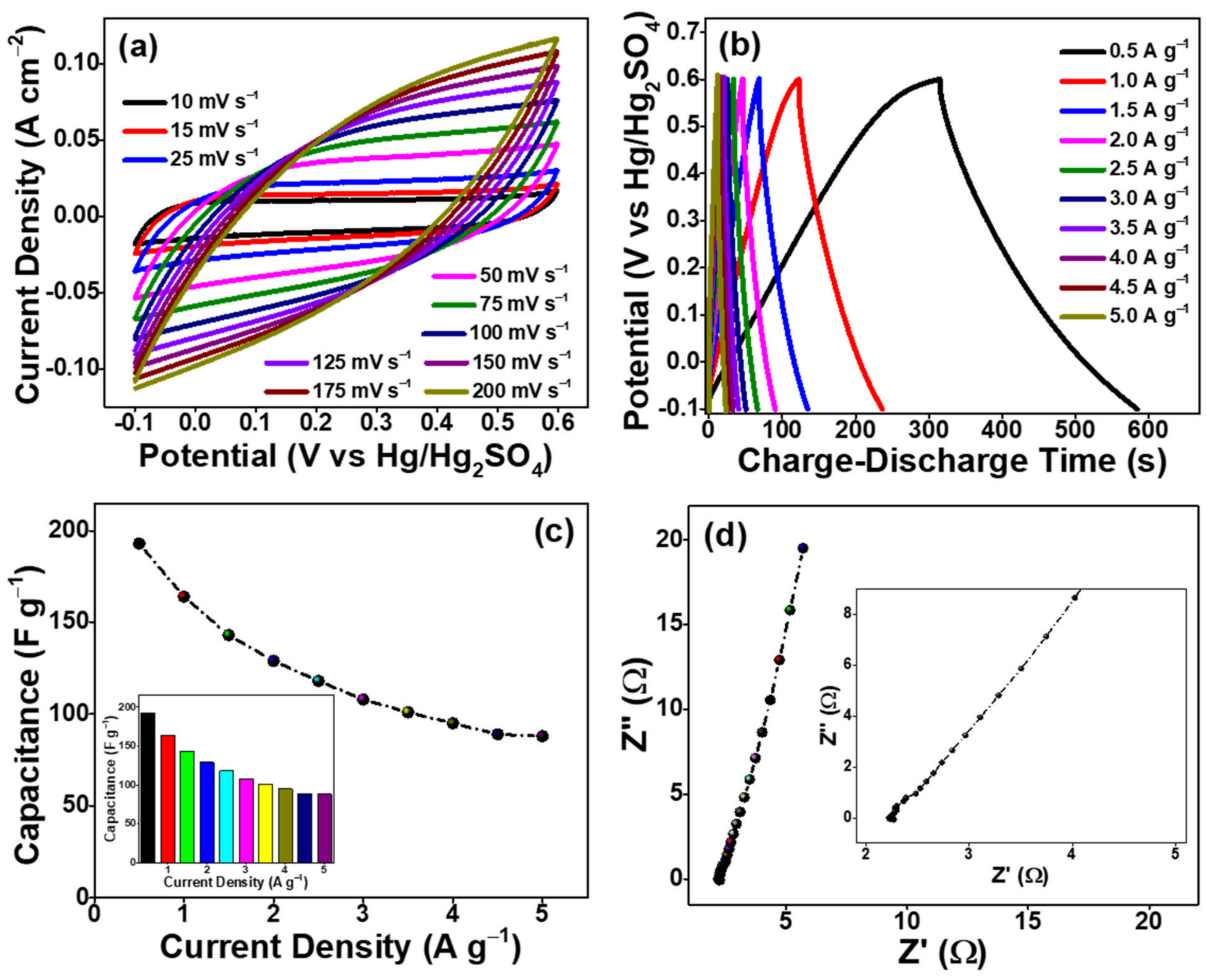
Electrochemical performance of as-synthesized AO-PC material in 1 M H_2_SO_4_ aqueous electrolyte. (**a**) CV curves when changing the scan rate from 10 to 200 mV s^−1^; (**b**) GCD curves when changing the current density from 0.5 to 5.0 A g^−1^; (**c**) specific capacitance when changing current density from 0.5 to 5.0 A g^−1^; and (**d**) EIS Nyquist plot with an alternating current amplitude of 5 mV (inset: magnified view of the high-frequency region of the EIS Nyquist plot).

A three-electrode configuration was used to examine the electrochemical performance of AO-PC material-based electrode in a 1 M H_2_SO_4_ electrolyte. Figure 6a shows the cyclic voltammetry curves of the AO-PC-coated carbon cloth (AO-PC/CC) electrode measured at scan rates between 10 and 200 mV s^−1^. The CV curves for the AO-PC/CC electrode present approximately rectangular shapes with some humps, indicating the coexistence of the electric double-layer (EDL) mechanism with insignificant pseudocapacitance. This quasi-rectangular shape is accredited to the presence of redox-active heteroatoms such as oxygen, sulfur, and nitrogen in the as-prepared AO-PC material [55]. In addition, it should be noted that the good quasi-rectangular shape below the scan rate of 100 mV s^−1^ indicates that the electrolyte ions have a rapid ionic response. The retention of a good quasi-rectangular shape signifies the material’s high rate capability and good capacitive behavior [29,56]. The CV curves become spindle-like shapes (fusiform) when the scan rate is increased to values greater than 100 mV s^−1^, indicating that the electrolyte cannot internally enter the AO-PC material. To further investigate the performance of the AO-PC material, GCD measurements were performed at different current densities in a 1 M H_2_SO_4_ electrolyte using a three-electrode configuration. The GCD curves of the AO-PC material at current densities varying between 0.5 and 5 A g^−1^ are presented in Figure 6b. The GCD curves of the AO-PC material show an approximately symmetrical triangular shape and faintly nonlinear slanted potential shapes with a higher current density, indicating EDL performance, fast charge–discharge response, low internal resistance, reversible adsorption–desorption of ions, and the occurrence of the redox reaction on the surface/edges of the AO-PC material-based electrode [56]. The redox reaction is caused by the heteroatom-containing functionalities, which can apparently improve the specific capacitance. At a current density of 0.5 A g^−1^, the GCD cycle was longer than at a current density of 5 A g^−1^. The specific capacitance was calculated at different current densities between 0.5 and 5 A g^−1^, as shown in Figure 6c. The specific capacitances of the as-prepared AO-PC material-based electrode were 193, 164, 143, 129, 118, 108, 101, 95, 89, and 100 F g^−1^ at the current densities of 0.5, 1.0, 1.5, 2.0, 2.5, 3.0, 3.5, 4.0, 4.5, and 5.0 A g^−1^, respectively (the inset in Figure 6c presents a bar chart of specific capacitances versus current densities) [57,58]. A capacitance retention of ~46% was observed, suggesting the good electrochemical performance (excellent rate capability) of the as-prepared AO-PC material. The limited fallout of capacitance might be due to the ingestion of the electrolyte and the irreversible reaction of the as-prepared AO-PC material-based electrode during the GCD process [59]. The greater quantity of charge storage in the as-prepared AO-PC material is due to the incorporation of sulfur and nitrogen atoms in the carbon framework [60]. 

**Figure 7:**
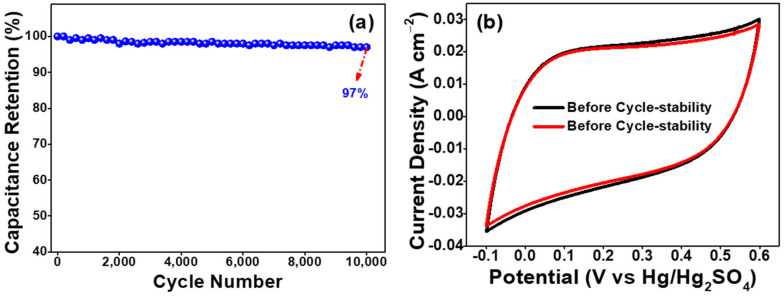
Electrochemical performance of the as-synthesized AO-PC material in 1 M H_2_SO_4_ aqueous electrolyte. (**a**) Cycle performance obtained by repeated GCD measurements at a current density of 5 A g^−1^ and (**b**) CV curves before and after cycle stability at a scan rate of 25 mV s^−1^.

EIS measurements were employed in the 0.01 Hz–10 kHz frequency range with an alternating current amplitude of 5 mV to further understand electrochemical performance. Figure 6d displays the EIS Nyquist plot of the as-prepared AO-PC material-based electrode. The Nyquist plot of the AO-PC material-based electrode is almost perpendicular to the real axis (Z′) in the low-frequency region, which indicates the ideal capacitive behavior and outstanding ion diffusion efficiency of the as-prepared AO-PC material. The outstanding ion diffusion might be due to the presence of the porous structure in the AO-PC material. In addition, it is composed of a quasi-semicircle in the high-frequency region (as is clear from the inset of Figure 6d), caused by the charge transfer resistance at the interface between the electrode and electrolyte [61]. The solution resistance and charge-transfer resistance of the AO-PC material-based electrode are around 2.2 Ω and 2.6 Ω, respectively. This low charge-transfer resistance is essential for improving the rate competence of supercapacitor materials, and can be attributed to the adequate pore structure of the electrode material; the promotion of ion transport and improved surface wettability of the electrolyte might be due to nitrogen and sulfur doping. Furthermore, the stability of the AO-PC material-based electrode was evaluated in a three-electrode configuration by continuous GCD measurement at a current density of 5 A g^−1^ in the potential window of −0.1 to 0.6 V. The capacitance retention of the AO-PC material-based electrode after 10,000 cycles is 97%, demonstrating excellent electrochemical stability (Figure 7a). After prolonged stability, CV and EIS measurements were conducted for the AO-PC material-based electrode and compared with the initial CV and EIS measurements to illustrate the strength of the as-prepared AO-PC material in acidic medium. The CV curves of initial and after-cycle stability for the as-prepared AO-PC material are shown in Figure 7b. The insignificant differences in the CV curves between initial and after-cycle stability suggest that the electrochemical process towards HER does not affect the structural stability of the as-prepared AO-PC material. Figure S4 shows the GCD curves and EIS Nyquist plots of the AO-PC material before (initial) and after cycling. It can be seen from the GCD curves and EIS Nyquist plots that there is an insignificant change in the properties. These results also indicate that the as-prepared AO-PC material is an outstanding electrode material for supercapacitor applications in an acidic medium. Finally, the obtained specific capacitance value was compared to previously reported biomass/biowaste-derived carbonaceous materials to show the superiority of the as-prepared AO-PC material. Table 1 demonstrates that the as-prepared AO-PC material has superior/comparable specific capacitance and outstanding durability compared to other biomass-/biowaste-derived carbonaceous materials. The remarkable electrochemical performance of the as-prepared AO-PC material might be due to the synergism between the presented heteroatoms and porous carbon framework in the AO-PC material.

## 4. Summary and Conclusions

A flexible working electrode was fabricated using the as-prepared AO-PC material as the electroactive material and 1 M H_2_SO_4_ as the aqueous electrolyte; its electrochemical performance was examined using CV, GCD, and cycle life measurements. The AO-PC material was derived from AO nut-skin waste as an inexpensive, environmentally friendly, and sustainable precursor through a direct pyrolysis route without using any chemicals or template. The structural characterization, including ATR-FTIR, XPS, and FE-SEM-EDX mapping, showed the presence of heteroatoms such as sulfur-, nitrogen-, and oxygen-bearing functional groups on the as-prepared AO-PC material. Nitrogen sorption studies revealed that the as-prepared AO-PC material possessed a surface area of 615 m^2^ g^−1^, with an average pore size of 3.5 nm. Heteroatoms possessing carbon and high surface area, and with adequate pores in the carbon framework, were correlated with enhanced conductivity of the as-prepared AO-PC material and improved wettability of the AO-PC material-based electrodes. In addition, the heteroatom and high surface area facilitated fast electronic transmission and increased the number of active sites. Because of these properties, at a current density of 0.5 A g^−1^, the as-prepared AO-PC material delivered a high specific capacitance of 193 F g^−1^. Note that the as-prepared AO-PC material exhibited 97% specific capacitance retention over 10,000 cycles at a current density of 5 A g^−1^. Based on the high capacitive behavior and low cost with facile fabrication of the heteroatom-doped biomass waste-based AO-PC material, it could be considered as an up-and-coming candidate for high-performance and sustainable energy storage applications.

## Figures and Tables

**Table 1:** Comparison of present results to the those in previously reported literature on biomass/biowaste-derived porous carbon towards supercapacitor applications.

Source (Biomass)	Preparation Method	Electrolytes	SC * (F g^−1^)/ CD * (A g^−1^)	Cycle/ Retention (%)	Reference
Succulent leaves	Carbonization	1 M H_2_SO_4_	128.4/1.0	-	[2]
Ginger straw	Annealing	EMIM-BF4	122/0.5	10,000/82.7	[19]
Garlic sprout	Carbonization	6 M KOH	568/0.1	5000/97	[20]
*A. turbinata*	Carbonization	1 M H_2_SO_4_	142/0.5	10,000/95	[62]
Areca palm	Carbonization	PVA–Li_2_SO_4_	132/0.5	5000/92	[27]
Kusha grass	Carbonization	6 M KOH	218/0.7	5000/88	[34]
Lotus fruit	Carbonization	1 M H_2_SO_4_	160/0.5	10,000/95	[63]
Hornbeam	Pyrolysis	1 M H_2_SO_4_	74/0.25	3500/99	[64]
Banana Peel	Carbonization	1 M H_2_SO_4_	137/0.5	10,000/94	[61]
AO Nut Skin	Pyrolysis	1 M H_2_SO_4_	193/0.5	10,000/97	This work

* SC—Specific Capacitance; CD—Current Density.

## Data Availability

Not applicable.

## Supplementary Materials

The following supporting information can be downloaded at: https://www.mdpi.com/article/10.3390/nano13101654/s1, materials, instrumentation methods, fabrication of working electrode and electrochemical measurements, structural characterization of as-prepared AO-PC material. Figure S1: Powder XRD pattern of as-prepared AO-PC material; Figure S2: Pore size distribution graphs of as-prepared AO-PC material; Figure S3: XPS survey spectrum of as-prepared AO-PC material; Figure S4: (a) GCD curves at a current density of 1 A g^−1^ before and after cycle-stability and (b) EIS EIS Nyquist plots at an alternating current amplitude of 5 mV before and after cycle-stability of as-prepared AO-PC material.
