# Supplementary material for: Facile Synthesis of Functionalized Porous Carbon by Direct Pyrolysis of Anacardium occidentale Nut-Skin Waste and Its Utilization towards Supercapacitors
Source: Nanomaterials (Basel). 2023 May 16;13(10):1654. doi: 10.3390/nano13101654 (PMC10224210; doi:10.3390/nano13101654)
Supplement: Supplementary file 1 [file nanomaterials-13-01654-s001.zip › nanomaterials-2373998-supplementary.pdf]

Supporting Information

Facile Synthesis of Functionalized Porous Carbon by Direct Pyrolysis of *Anacardium Occidentale* Nut-Skin Waste and Its Utilization towards Supercapacitors

Raji Atchudan [1,*,†], Suguna Perumal [2,†], Ashok K. Sundramoorthy [3,†], Devaraj Manoj [4,5,†], Raju Suresh Kumar [6], Abdulrahman I. Almansour [6] and Yong Rok Lee [1,*]

1 School of Chemical Engineering, Yeungnam University, Gyeongsan 38541, Republic of Korea
2 Department of Chemistry, Sejong University, Seoul 143747, Republic of Korea; suguna.perumal@gmail.com
3 Department of Prosthodontics, Saveetha Dental College and Hospitals, Saveetha Institute of Medical and Technical Sciences, Poonamallee High Road, Velappanchavadi, Chennai 600077, Tamil Nadu, India; ashok.sundramoorthy@gmail.com
4 Department of Chemistry, Karpagam Academy of Higher Education, Coimbatore 641021, Tamil Nadu, India; manojdvrj@gmail.com
5 Centre for Material Chemistry, Karpagam Academy of Higher Education, Coimbatore 641021, Tamil Nadu, India
6 Department of Chemistry, College of Science, King Saud University, Riyadh 11451, Saudi Arabia; sraju@ksu.edu.sa (R.S.K.); almansor@ksu.edu.sa (A.I.A.)
* Correspondence: atchudanr@yu.ac.kr (R.A.); yrlee@yu.ac.kr (Y.R.L.)
† These authors contributed equally to this work.

Materials

The dried *Anacardium occidentale* nut's skin waste was collected from Tamil Nadu, India. N-Methyl-2-pyrrolidone (NMP), polyvinylidene fluoride (PVDF), and sulfuric acid (H_2SO_4) were purchased from Sigma-Aldrich, Seoul, Republic of Korea. Carbon cloth (CC) electrode was purchased from FuelCellStore, College Station, Bryan, TX, USA. Nitrogen gas was obtained from Duksan, Seoul, Republic of Korea. All the chemicals were used as purchased, the deionized (DI) water was used throughout this study.

Instrumentation methods

The prepared functionalized porous carbon from dried *Anacardium occidentale* nut's skin (AO-PC) was characterized by various physicochemical techniques such as field emission scanning electron microscopy (FESEM, Urbana, IL, USA) with energy-dispersive X-ray spectroscopy (EDS), high-resolution transmittance electron microscopy (HRTEM, Peabody, MA, USA), Raman spectroscopy, nitrogen adsorption-desorption isotherms, attenuated total reflectance Fourier transform infrared (ATR-FTIR) spectroscopy, and X-ray photoelectron spectroscopy (XPS). FESEM with EDS analysis was carried out on a Hitachi S-4800 equipped with EDX at an accelerating voltage of 4 kV. TEM/HRTEM images were performed with a JEOL JEM transmission electron microscope with an operating accelerating voltage of 120 kV. Raman spectrum was recorded on XploRA Micro-Raman spectrophotometer (Horiba, Palaiseau, France) with the range between 50 and 4000 cm^{-1} at the core research support center for natural products and medical materials of Yeungnam University. Nitrogen physisorption isotherms were measured at −197 °C using Micromeritics ASAP 2000 (Norcross, GA, USA). Before the experiments, the samples were dried at 120 °C and evacuated for 8 h in flowing argon at the flow rate of 60 standard cubic centimeters per minute at 140 °C. Surface area, pore size, and pore volumes were obtained from isotherms using the conventional Brunauer-Emmet-Teller (BET) and Barrett-Joyner-Halenda (BJH) equations. ATR-FTIR spectra were recorded in transmittance mode on a Perkin Elmer Spectrum Two (Raymond Ave., Fullerton, CA, USA) in the wavenumber range from 400 to 4000 cm^{-1} by the addition of 8 scans at a resolution of 8 cm^{-1}. XPS spectra were achieved using a K-Alpha (Thermo Scientific, Thermo Scientific, Seoul, South Korea). CasaXPS software was used for the deconvolution of the high-resolution XPS spectra.

Fabrication of working electrode and electrochemical measurements

The prepared AO-PC was used for the fabrication of the working electrodes. AO-PC and polyvinylidene fluoride (PVDF) with the wt. % of 95 and 5 were ground well in an appropriate amount N-Methyl-2-Pyrrolidone (NMP) to make a homogeneous paste for the fabrication of the working electrode, respectively. The resulting homogeneous paste was drop-casting on the carbon cloth (CC) with an area of 1 cm^2, and sequentially the electrode was kept at 100 °C in a hot air oven for 24 h to dry the electrodes. After the fabrication, the obtained modified working electrodes were examined for hydrogen evolution reaction (HER) performance. All electrochemical measurements, including cyclic voltammetry (CV), galvanostatic charge-discharge (GCD), and electrochemical impedance spectroscopy (EIS) were conducted with a typical three-electrode system and were performed on the CorrTest-CS350 electrochemical workstation in 1.0 M H_2SO_4 solution. A commercial Hg/Hg_2SO_4 (Sat. K_2SO_4) electrode was employed as a reference electrode, a platinum plate (1 cm^2) was used as the counter electrode, and AO-PC coated CC (AO-PC/CC) was used as working electrodes, respectively. The CV measurements were carried out at a potential window between –0.1 and 0.6 V (vs. Hg/Hg_2SO_4) under the different scan rates from 10 to 200 mV s^{-1}. The GCD measurements were performed with a potential window between –0.1 and 0.6 V (vs. Hg/Hg_2SO_4) at the current densities varied from 0.5 to 5.0 A g^{-1}. EIS measurements were performed in the frequency range of 0.01 Hz–10 kHz with an alternating current amplitude of 5 mV. All the electrochemical tests were conducted at room temperature. The specific capacitances of the electroactive materials were obtained from their GCD curves according to the following equation (S1).

$$C_s = \frac{I\Delta t}{m\Delta V} \tag{S1}$$

whereas Cs is the specific capacitances, I is the current in the charge-discharge process (A), Δt represents the discharge time (s), ΔV stands for the potential window during the charge-discharge measurement (V), and m donates the mass of the electroactive materials (g).

Structural Characterization of As-Prepared AO-PC Material

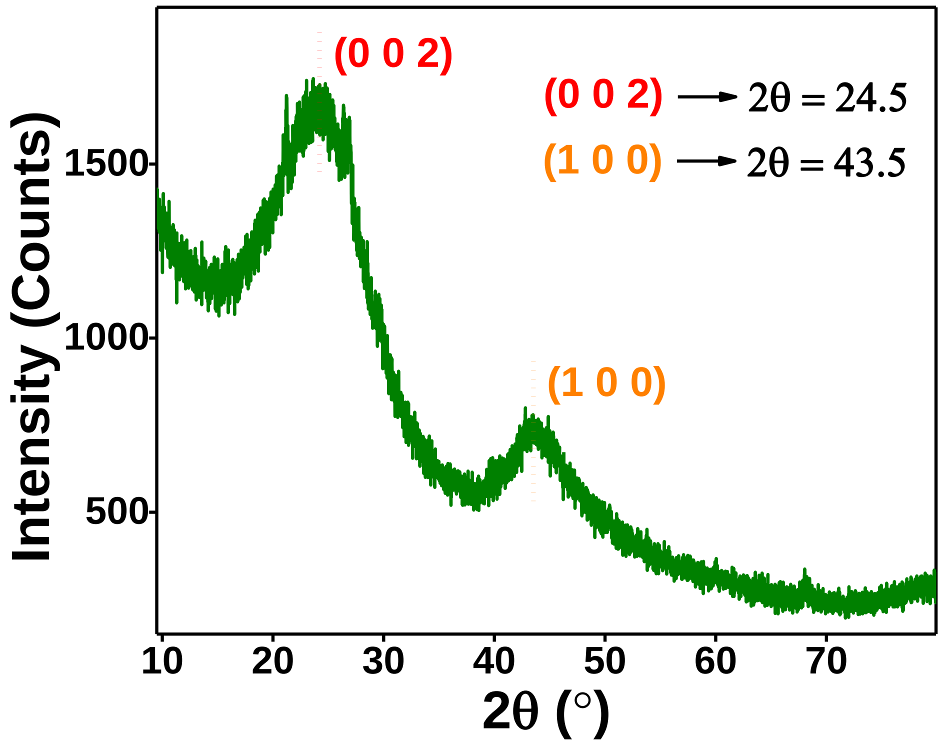

Figure S1. Powder XRD pattern of as-prepared AO-PC material.

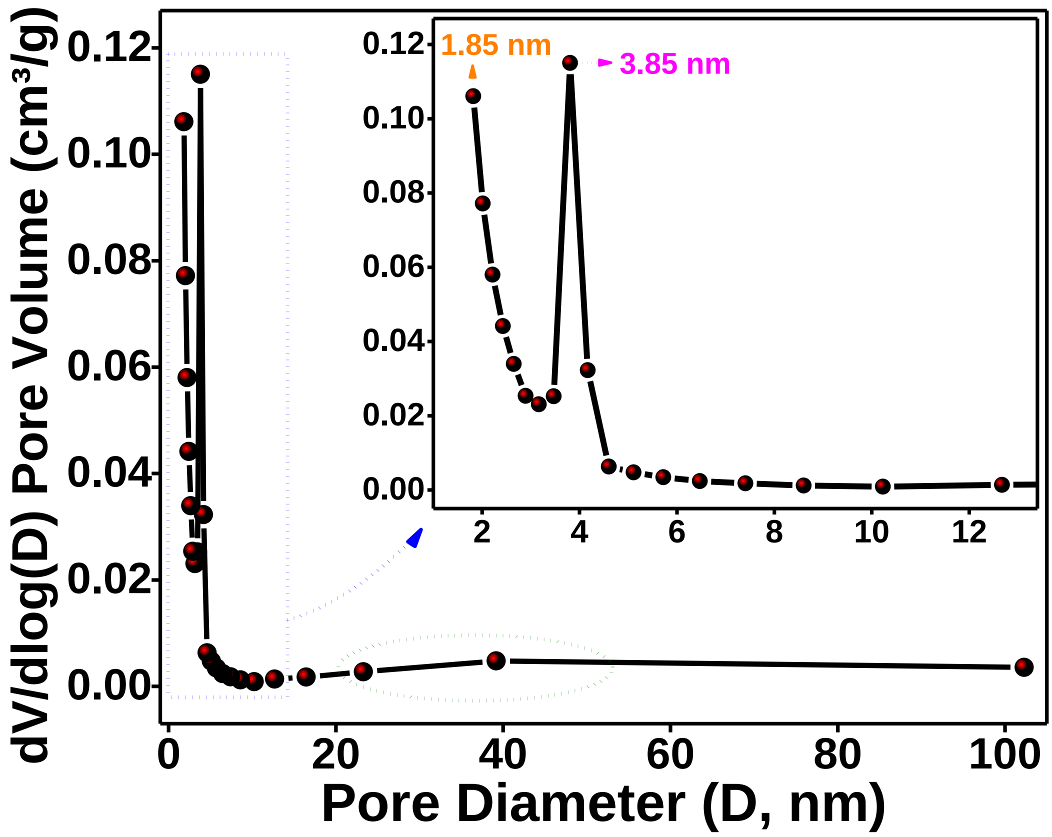

Figure S2. Pore size distribution graphs of as-prepared AO-PC material.

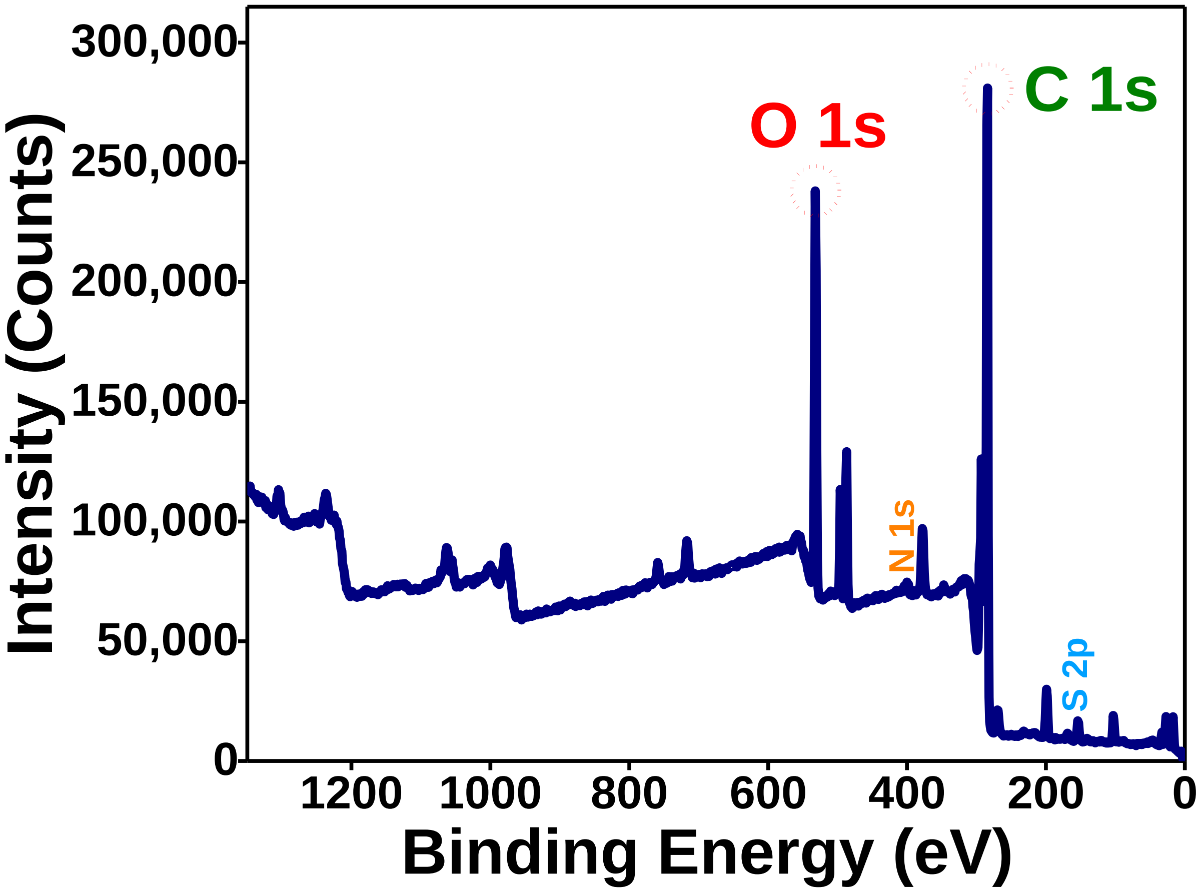

Figure S3. XPS survey spectrum of as-prepared AO-PC material.

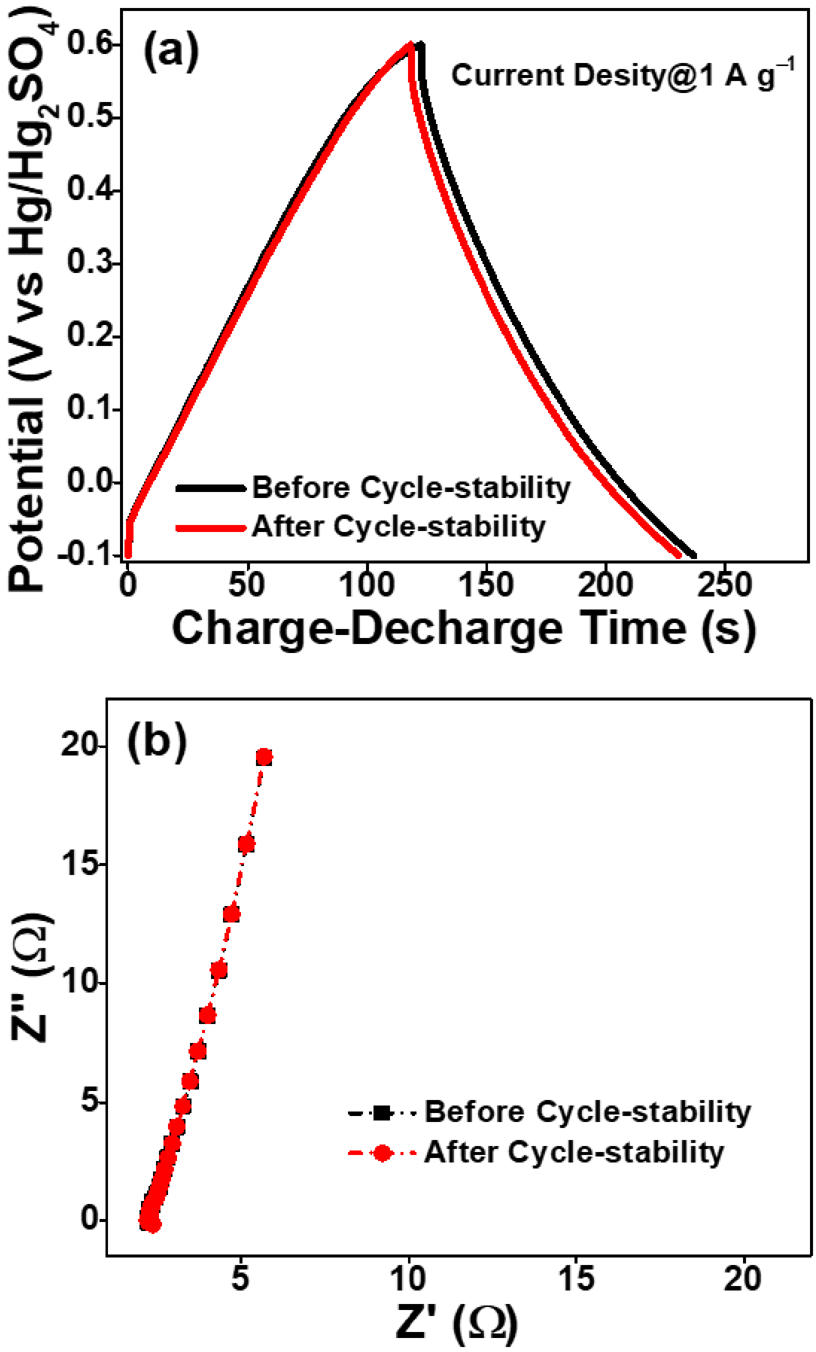

Figure S4. (**a**) GCD curves at a current density of 1 A g^{-1} before and after cycle-stability and (**b**) EIS Nyquist plots at an alternating current amplitude of 5 mV before and after cycle-stability of as-prepared AO-PC material.
